# Post-test adverse psychological effects and coping mechanisms amongst HIV self-tested individuals living in couples in urban Blantyre, Malawi

**DOI:** 10.1371/journal.pone.0217534

**Published:** 2019-06-12

**Authors:** Moses Kelly Kumwenda, Elizabeth Lucy Corbett, Augustine Talumba Choko, Jeremiah Chikovore, Kruger Kaswaswa, Mphatso Mwapasa, Rodrick Sambakunsi, Tore Jarl Gutteberg, Stephen Gordon, Alister Munthali, Nicola Desmond

**Affiliations:** 1 Malawi Liverpool Wellcome Trust, Blantyre, Malawi; 2 Helse Nord TB initiative, College of Medicine, Blantyre, Malawi; 3 Clinical Research Department, London School of Hygiene and Tropical Medicine, London, United Kingdom; 4 Infectious Disease Epidemiology Department, London School of Hygiene and Tropical Medicine, London, United Kingdom; 5 Social Aspects of Public Health Research Programme, Human Sciences Research Council, Durban, South Africa; 6 Population Health Department, London School of Hygiene and Tropical Medicine, London, United Kingdom; 7 University of Tromso, The Arctic University of Norway, Tromsø, Norway; 8 University Hospital of North Norway, Tromsø, Norway; 9 Clinical Sciences Department, Liverpool School of Tropical Medicine, Liverpool, United Kingdom; 10 Centre for Social Research, Chancellor College, Zomba, Malawi; Lluita contra la SIDA Foundation - Germans Trias i Pujol University Hospital - Autònoma de Barcelona University, SPAIN

## Abstract

**Introduction:**

Mandatory face-to-face counselling is necessary during HIV testing but difficult to implement within the context of HIV self-testing. We investigated adverse psychological effects and coping mechanisms following HIV-positive and HIV-discordant test results amongst self-tested individuals living in couples in urban Blantyre, Malawi.

**Methods:**

Qualitative data from 35 in-depth interviews with self-tested individuals living in couples for more than 3 months were collected and analysed using thematic content analysis.

**Results:**

Adverse psychological effects seemed to mostly occur among individuals learning for the first-time that they were HIV-positive or living in HIV-discordant relationship. Irrespective of test outcomes, women living in couples expressed difficulty making important decisions about the future of their relationships while men seemed to shoulder the emotional burden associated with feeling or being seen as responsible for introducing HIV into the relationship. Post-test psychosocial support and ascertained positive behaviour change of the perceived index partner allowed some couples to overcome adverse psychological effects linked to test results.

**Conclusion:**

Self-tested individuals living in couples may lack collective coping capability to collaboratively manage post-test adverse events after new HIV-positive or HIV-discordant results. Psychosocial support seemed to enable couples to foster both an individual and a collective ability to manage adverse psychological effects within the context of a couple. More research is needed to ascertain the magnitude of the deficiency of collective coping competency in couples following an HIV test.

## Introduction

HIV self-testing (HIVST) is a novel approach to HIV testing that has great potential for reaching under-served groups of people such as men, youth and key populations such as female sex workers and men who have sex with men (MSM). HIVST occur when “an individual collects his or her own sample; performs a simple, rapid non-laboratory test; and is the first to know ones results” [1]. Blood-based and oral fluid-based test-kits options for HIVST are available with the latter having more empirical evidence on feasibility, acceptability and accuracy than the former [2,3]. Allowing individuals to perform their own HIV test introduces certain worries including a potential for abuse, for instance, through use on others people without the requisite consent, or through coercing partners in sexual relationships into testing [4]. There are also concerns about the potential for adverse psychological effects following HIV-positive results and the problem of managing such effects especially when the test has been carried out in absence of a trained professional [5].

Mandatory face-to-face counselling–contained within the global normative guidelines on HIV testing [6]—is key in the management of adverse psychological effects and an important feature of a conventional HIV testing process. A key criticism against HIVST is the absence of face-to-face counselling since by definition, the counsellor or third parties are eliminated from the testing context and so delinking the HIV testing process from any potential HIV counselling. Without direct supervision by trained professionals, HIVST can result in testing without reporting results to public health authorities, failure to receive confirmatory testing, inadequate linkage to treatment for individuals who test HIV-positive, and testing without partner consent/notification in couples [7]. The 2016 HIV Testing Services (HTS) guidelines for Malawi list consent among five key principles guiding the provision of HIV testing services, and pre- and post-test counselling as an integral to the testing process [8]. In the standard model, HIV counselling is delivered by trained and certified counsellors who provide information that may help individuals manage test-related adverse psychological effects. Thus, post-test counselling given to clients is aligned with the test result and is packaged with information about HIV risk and risk reduction, the meaning of test results, linkage to relevant services and disclosure.

Expanding access to HIV testing services to couples and cohabiting partners is an important step towards prevention and treatment since HIV acquisition through heterosexual contacts is high in this group [9,10]. However, the organisation of routine HIV testing services usually fails to attract both partners to test together [11] with unacceptably high levels of non-uptake of testing among male partners [12]. There has been limited emphasis on couple-oriented HIV-related programmes partly because implementing such strategies is logistically daunting and ethically complex [13,14]. The process of testing couples and ensuring serostatus disclosure is also intricate, remains fraught with anxiety and is particularly challenging for HIV-positive women [15]. Concerns remain prevalent over negative consequences following couples testing and HIV serostatus disclosure to a partner which are largely driven by anticipated stigma, fear of intimate partner violence, rejection, loss of intimacy and threats to one’s status and standing within the relationship [16,17].

Research demonstrates that positive psychological approaches contained within post-test counselling improve general wellbeing of couples by enhancing resilience when encountering adverse events and fosters effective management of HIV infection and relationship dynamics [18]. Implementing CHTC may also lessen the burden of disclosing one’s HIV-positive serostatus to a partner and addressing existing power imbalances within sexual relationships [19]. Currently, no data is available regarding HIV serostatus disclosure being facilitated by HIVST in couples, but also about post-disclosure dynamics within sexual relationships. Despite a provision of counselling in policy documents guiding HIV testing processes, there is no guarantee that such counselling fully addresses adverse psychological effects related to disclosure of serostatus to a partner. Furthermore, the actual manifestation of adverse psychological effects in couples linked to HIVST has not been ascertained. We investigated adverse psychological effects after HIV-positive concordant or HIV-discordant results amongst self-tested individuals living in couples.

## Methods

### Ethical considerations

College of Medicine Research Ethics Committee (COMREC) approved this study (P.05/12/1211). Participation in this research was voluntary and a written informed consent was obtained from all study participants. Blantyre City Assembly and Blantyre District Health Office provided permission to conduct field work.

### Theoretical framework—The Self-efficacy theory

Diagnosis of potentially life-threatening illness such as HIV could be an experience that may trigger adverse psychological effects that manifest in various forms including impaired concentration, depression, sleep disturbances, self-devaluation, emotional detachment from others and disengagement from meaningful aspects of life [20–22]. Adverse psychological effects in this study were defined as an uncomfortable feeling towards HIV-positive or HIV-discordant self-test results and could include nervousness or depression. How individuals recover after learning for the first-time that they are HIV-positive or are living in an HIV-discordant relationship may help us understand the immediate post-test reaction and how individuals or sexual partners as a collective unit overcome HIV test-related adverse psychological effects. We employed the self-efficacy theory framed within the social cognitive theory to understand the post-test reactions and coping competency after HIV-positive and HIV-discordant self-test results within a context of individuals living in heterosexual relationships. Self-efficacy is defined as “an individual’s own perceived ability to manage psychological and environmental demands associated with stressful events” [22]. ‘Coping ability’ in this paper is defined as the capacity to successfully navigate and adequately manage feelings after a HIV-positive and HIV-discordant self-test results.

### Setting

Baseline data were collected within a qualitative study exploring the long-term consequences of semi-supervised HIVST on partnership dynamics amongst heterosexual couples. This qualitative study was nested within a cluster randomized trial (CRT) investigating the impact of intensified HIV/TB prevention on the incidence of bacteriologically confirmed TB implemented in high-density slums (Ndirande, Chilomoni, and Likhubula) of urban Blantyre, Malawi—ISRCTN02004005 [23]. Through resident community counsellors (CCs) trained in HIV Testing and Counselling (HTC) by Ministry of Health (MoH), the CRT offered HIVST to adult community members using OraQuick_ ADVANCE I/II (Orasure Inc.–assembled in Thailand for OraSure Technologies, Bethlehem, PA). CCs provided adult community members interested to self-test with a demonstration of using the self-test kit followed by a brief test of competency to understand instructions and independently perform a self-test without supervision. Those competent were allowed to self-test at home and given the option to voluntarily disclose self-test results to the CCs. Self-tested individuals who disclosed to CCs were provided appropriate post-test counselling and referral to follow-up healthcare when eligible while those who did not disclose were provided with post-test counselling for both HIV-positive and HIV-negative results. In addition, the CCs were emphatic about the importance of a trained counsellor confirming all HIV-positive self-test results.

### Participant selection

Partners who had self-tested together as a couple or where at least one partner had self-tested and community members living in the CRT catchment area were recruited in the qualitative study [24]. We conducted 35 in-depth interview (IDIs) with participants (Female N = 18) who obtained either HIV-positive or HIV-discordant self-test results living in sexual relationships. Demographic characteristics of study participants are provided in Table 1. We included participants who were 18 years or older, in a relationship of more than 3 months, had self-tested within 2 weeks, had provided an oral consent to be approached at home, were willing to provide an informed consent, to disclose HIV serostatus to the researcher and to undergo a confirmatory self-test. With help from the CCs, we identified and recruited self-tested persons who provided an initial oral consent to be approached at home from the CRT’s HIVST registers (see Table 1).

**Table 1:** Demographic characteristic of IDI participants.

Categories		Sex	
		Male	Female
		17	18
**Age**	Range	22–61	19–45
	Average (in years)	35.3	28.7
**Education**	No education	0.0%	0.0%
	Primary	35.3%	55.6%
	Secondary	58.8%	44.4%
	Tertiary	5.9%	0.0%
**Occupation**	Unemployed	0.0%	66.7%
	Informal	82.4%	11.1%
	Formal	17.6%	22.2%
**HIV Status**	HIV-positive	14*	14**
	HIV-negative in HIV-discordant relationship	3	4

*Out of the 14 HIV-positive men, 12 had children (Range = 1–4 children)

** All the 14 HIV-positive women had children (Range = 1–6 children)

Purposive sampling was used to provide maximum variation of study participants in order to have a wide-ranging spectrum of attributes and maximise representation within pre-selected categories [25]. The sampling attributes of variation for participants were sex (male and female) and HIV serostatus of the relationship (HIV-positive concordant and HIV-discordant result). Eight out of 28 HIV-positive study participants already knew that they were infected with HIV before self-testing and were included in our sample because previous knowledge of HIV-positive serostatus was not among study’s exclusion criteria. We have described reasons influencing individuals who already knew about their HIV-positive serostatus before self-testing in a previous paper [24].

Trained qualitative researchers—Moses Kumwenda (MK), Mackwelling Phiri (MP), and Daniel Mwale (DM)—prospectively identified study participants from the CRT HIVST registers within 14 days of self-testing. Identified individuals were physically approached and contacted with the help of CC’s. Study participants did not know the interviewer prior to the interview. No participant who was approached to participate in this study refused participation and all participants were interviewed at their homes. For ethical reasons and to enhance active participation of participants, sexual partners in a couple were interviewed separately. Non-participants were not allowed to be present during qualitative interviews.

### Data collection

Semi-structured guides steered interviews using Chichewa, a dominant language in the study setting. Topic guides explored topics about adverse psychological effects after self-testing HIV-positive/HIV-discordant such as feelings, dislikes, worries, disappointments and regrets. Additional probes were included in our topic guides to understand how individuals or couples navigated post-test problems. Audio recorders were used to capture qualitative data and the duration of interviews ranged between 27 minutes and 1 hour 10 minutes. Interview duration depended on how different participants responded to questions. Collected data were transcribed verbatim and cleaned by trained transcribers–Ruth Maonga (RM) and reviewed for accuracy by MK. In addition, summative field notes were made after each interview and were very useful in providing quick impressions of emerging themes.

### Data analysis

Completed transcripts were imported into NVIVO 9 QRS software (QSR, Melbourne, Australia) for organisation, management and analysis [26]. To prevent diluting meanings from transcript, transcribed data were coded by two coders (i.e. MK and MP) and analysed directly from Chichewa by MK, MP, Augustine Choko (AC), Mphatso Mwapasa, Rodrick Sambakunsi (RS) and Kruger Kaswaswa (KK) who are Chichewa speakers. Translation from Chichewa to English occurred during data analysis. Thematic content analysis was used to analyse data because it is suitable for simple reporting of common issues mentioned in data when conducting exploratory research on a subject where not much is known [27]. Transcribed data were inductively and deductively coded by carefully reading and re-reading transcripts, identifying emerging codes from the data but also deriving codes from the self-efficacy theoretical framework to guide the discussion of the results [28]. Codes were then marked with unique descriptive identifying words or category names. During data collection and coding, it was determined that saturation of information had been attained when emergent themes and codes began to appear recurrently [29]. Data were triangulated and validated across couples and by comparing analytical segments between different groups of participants. Deductive and inductive codes were reviewed to identify intersections and to group them into categories.

## Results

**Fig 1:**
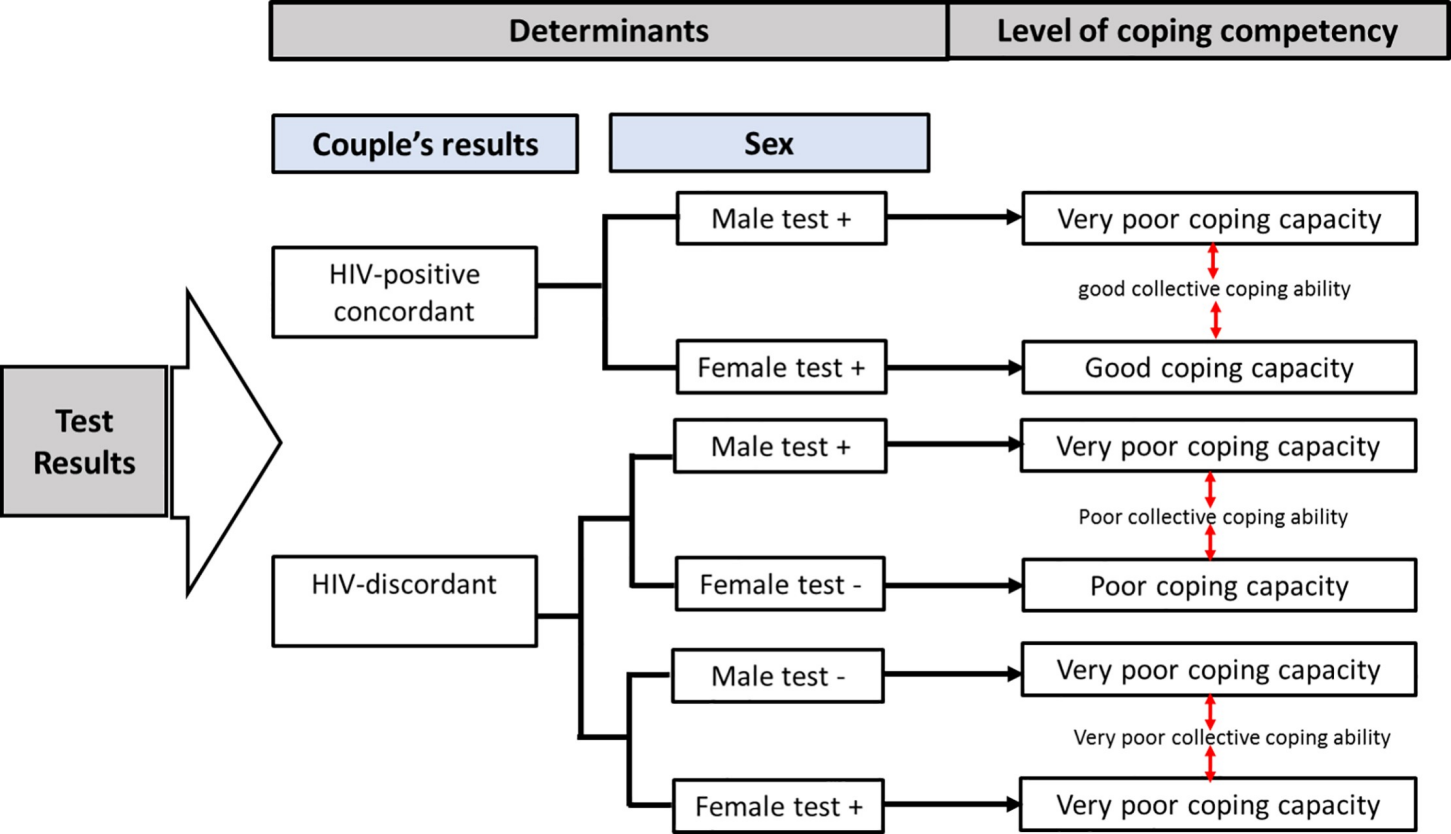
Individual and partners collective coping competency after HIV-positive and HIV-discordant test results. test + = test HIV-positive; test— = test HIV-negative.

Demographic characteristics of study participants (Table 1) illustrate that male participants were older, better educated and more engaged in both formal and informal income generating activities than female participants. Study results from our qualitative analysis demonstrate presence of adverse psychological effects related to HIV-positive and HIV-discordant self-test results. Fig 1 shows post-test reactions following new HIV-positive and HIV-discordant results in relation to the level of coping competency while Table 2 contains quotes from participants on key themes.

**Table 2:** Quotes on adverse psychological effects and coping abilities.

Theme	No.	Quote
**Adverse psychological effects among individuals living in HIV-discordant couples**	Q1	*“When I sat quietly at home on the day that we tested*, *my wife asked*, *‘what are you thinking about*?*’ I responded*, *‘do you think that I am contemplating about our results*.*’ She told me that she asked about this because she was worried that I could abandon the marriage*.*”* (Male, 23 years, HIV-negative, Discordant)
	Q2	*“I did not believe that this can be possible—all these years that I have been with her without telling her that I am positive*. *I said that ‘no*, *let us try another [testing] approach* .* *.* *. *to be sure this is true’*.*”* (Male, 43 years, HIV-positive, Discordant)
	Q3	*“How have you have been found like this [HIV-positive]*? *… how is it possible that this disease is now found in our marriage when I never had sexual intercourse with any other man*?*”* (Female, 37 years, HIV-negative, Discordant)
**Adverse psychological effects among individuals living in HIV-positive concordant couples**	Q4	*“I felt very sad about my situation that I have HIV considering that I may die soon*. *I was heartbroken*, *and I felt that I may die as a result of this disease while I have little children that could be orphaned whilst very young*.*”* (Female, 27 years, HIV-positive, Concordant)
	Q5	*“I was gripped with fear about how he would react if I tell him that I have HIV*. *I was worried how we will live together in this relationship*.*”* (Female, 38 years, HIV-positive, Concordant).
	Q6	*“I was happy that we knew that we both have HIV in this house*. *We knew about this together*, *and this removed a chance that one person would blame the other*. *We just accepted that we are an HIV-positive couple who would like to have a happy life*.*”* (Female, 31 years, HIV-positive, Concordant)
	Q7	*“After self-testing*, *we did not regret anything when we knew that we are HIV-positive*. *We do not have any worries right now and we live the way we used to before testing*.*”* (Male, 31 years, HIV-positive, Concordant)
	Q8	*“We just accepted that we are both positive*. *My wife did not complain or get disappointed*. *She did not do anything but just accepted that we are infected with HIV*. *It was not difficult for her to accept this*.*”* (Male, 46 years, HIV-positive, Concordant)
	Q9	*“Although we were disappointed a little at first*, *we were also happy on the other hand that it was not a problem because I have HIV and so does my wife*. *We should just play to God to give us both a long life*.*”* (Male, 47 years, HIV-positive, Concordant)
***Sex-based differences in adverse psychological effects***	Q10	“*I cannot divorce him because I think about the future of my children*. *If I end this marriage*, *I will put my children in trouble*. *The woman that my husband may marry after me cannot take good care of my children*. *I cannot also provide everything that the children need*.*”* (Female, 37 years, HIV-negative, Discordant)
	Q11	*“I see that he thinks a lot these days*. *When I speak to him*, *it seems as if he is not concerned*. *Nowadays when he comes back from work*, *he only plays with our child*. *In the past*, *he used to tell me many things that he had encountered*, *and we used to laugh*, *but now* .* *.* *. *he just stays quiet*.*”* (Female, 28 years, HIV-positive, Discordant)
	Q12	*“I now feel sorry about myself because if this HIV*. *In all my youth*, *I was very careful until I got married to this man*. *I was fine [HIV-negative] all along until now*.*”* (Female, 24 years, HIV-positive, Concordant
	Q13	*“I regretted being in this relationship because we both tested HIV-negative before*. *I even told my sister that I am divorcing this man*, *but she said how can I divorce him when he has already damaged you*?*”* (Female, 42 years, HIV-positive, Concordant)
	Q14	*“My friend [spouse] may know something about this [HIV infection] since he is a man and knows his conduct [sexual misconduct]*. *But for me*, *I have always lived without having boyfriends*. *But for a man*, *you cannot know how he behaves and what he does at his workplace*. *Since he never said anything [after testing HIV-positive]*, *he definitely knew something about this*. *I think he is the one who infected me*.*”* (Female, 38 years, HIV-positive, Concordant)
	Q15	*“I trust my wife*. *I do trust that she does not have other sexual partners… because she now knows Jesus…”* (Male, 30 years, HIV-positive, Concordant)
	Q16	*“I do not believe that it is my wife who has infected me because she has always been faithful to me and there is nothing suspicious that she has done in this relationship*.*”* (Male, 31 years, HIV-positive, Concordant)
***Positive coping mechanisms for overcoming test-related adverse psychological effects***	Q17	*“I trust him a lot now*. *The counselling we received after self-testing has brought encouragement that we should be trusting each other and that one of us should not go elsewhere [should not seek other sexual partners]*.*”* (Female, 26 years, HIV-positive, Concordant)
	Q18	*“For me*, *our relationship has been very good*. *I found that after receiving counselling by that friend of yours [member of the study team]*, *we were encouraged*, *and our love was strengthened*. *We now love each other very much*.*”* (Female, 28 years, HIV-positive, Discordant)
	Q19	*“He has stopped moving about [having other sexual partners]*, *even the alcohol that he used to drink*, *he stopped drinking these days*. *He does not appear to be having an affair [extramarital relationships] these days*.*”* (Female, 37 years Female, HIV-negative, Discordant)
	Q20	*“Most of the times we are together at home … It is not often that he goes out even during the weekend*. *When he comes from work*, *we are together … there has been nothing suspicious that he had done these few days*.*”* (Female, 37 years Female, HIV-negative, Discordant)
	Q21	*“I used to be unhappy most of the times … I was getting sick because of thinking too much that he might find other women*, *and this means that I would suffer*. *But now I do not think about that because we live trusting and loving each other*.*”* (Female, 28 years, HIV-positive, Discordant)
***Negative coping mechanisms for overcoming test-related adverse psychological effects***	Q22	*“I went to test again (at a clinic) to find the truth because I know that HIV is found in blood and not saliva*. *It was difficult for me to believe that the results that I got using this device (HIVST) were real*.*”* (Male, 30 years, HIV-positive, Concordant)
	Q23	*“I have tested several times and they still find that I am infected*. *That she does not have it [HIV virus]*, *it is not true*. *I do not really understand that this is possible*. *Maybe she self-tested wrongly*.*”* (Male, 43 years, HIV-positive, Discordant)
	Q24	*“I do not have any trust that maybe I am truly negative or positive*, *I still live with uncertainty about my HIV status*.*”* (Female, 19 years, HIV-negative, Discordant)
	Q25	*“There were question marks because we were different [HIV-discordant]*. *I failed to understand how this was ‘thekable’ [possible]*. *I sometimes ask myself that possibly it is these sachets [locally distilled rum] that constantly burst [breaks and drink] that have burnt the virus inside me*?*”* (Male, 32 years, HIV-negative, Discordant)
	Q26	*“It is not through infidelity*, *razors or needle that we have HIV*, *no*. *The only answer is* .* *.* *. *that witches deliberately infected us with AIDS*.*”* (Male, 31 years, HIV-positive, Concordant)
	Q27	*“… when I look at you now [male partner]* .* *.* *. *I see you as a monster because you have damaged my body [infected her with HIV]”* (Female, 24 years, HIV-positive, Concordant)

### Adverse psychological effects among individuals living in HIV-discordant couples

Study participants learning for the first-time about their HIV-positive serostatus or being in HIV-discordant relationship were greatly shocked. In contrast, only a few study participants who previously knew they were HIV-positive were worried, possibly because they had developed coping mechanisms following an initial diagnosis and had already adapted to their HIV-positive serostatus. An instantaneous response to new HIV-positive HIVST results was characterised by immense distress primarily because of the fear of death.

Test-related worries were more noticeable and amplified amongst individuals living in HIV-discordant couples than individuals living in HIV-positive concordant couples, ostensibly because HIV-discordant results were not anticipated and emerged when the client was unprepared to face such test outcomes (Table 2; Q1). Quote 1 supports a popular view regarding the prospect of marriage disintegration after HIV-discordant results due to a fear of death. Among individuals living in HIV-discordant couples, partners mutually wondered just how one partner was not infected with HIV after numerous exposures to probable HIV infection through unprotected sexual intercourse (Table 2; Q2). Quote 2 illustrates that when self-testers doubted HIVST results, it prompted them to have the results confirmed through routine HIV testing facility.

Some HIV-negative partners in HIV-discordant couples wondered exactly how their HIV infected partner contracted the virus (Table 2; Q3). Quote 3 depicts elements of infuriation of an HIV infected woman towards her male partner that emerged after self-testing. In the quote, the woman was disturbed emotionally when she learned that her husband was HIV-positive.

### Adverse psychological effects among individuals living in HIV-positive concordant couples

Like individuals with HIV-discordant results, those learning for the first-time that they were concordant HIV-positive exhibited characteristics suggestive of post-test emotional turmoil. In this case, blame and distrust was directed towards the male partner or a partner with a pre-existing record of infidelity. Individuals learning for the first-time that they were infected with HIV felt angry and sorry for themselves and seemed less able to deal with both the knowledge of having the infection and their relationship. HIV-positive individuals living in HIV-positive concordant relationships were mostly worried that they could died soon because of HIV. Women were mostly concerned that if they die early, no one would be able to fend for their young children the way they do it (see Table 2; Q4). Individuals were also deeply terrified about how the partners would react when notified about an HIV-positive result and subsequent scepticism about survival of the relationship as illustrated by Q5 in Table 2. This was particularly a big concern when the initial self-test was conducted in absence of a sexual partner.

Unlike in those in HIV-discordant relationships, individuals whose self-test results were concordant with those of the partner were quick to collectively address their post-test worries possibly because of having a shared HIV serostatus. Both men and women indicated that they easily navigated their situation and quickly recovered from test-related emotional distress (see Table 2; Q6 and Q7). Knowledge that both partners were infected was liberating to some individuals and learning together through HIVST at home made it easier for partners to accept their situation and to look for appropriate follow-up medical attention (see Table 2; Q8 and Q9).

### Sex-based differences in adverse psychological effects

HIV-discordant results were disappointing to both partners but were more disconcerting to male partners where the female partner was the one who tested HIV-positive. Out of the 14 participating individuals living in HIV-discordant couples (i.e. 7 couples), two couples (out of three in which the index partner was a woman) divorced within three months after HIVST. The third couple nearly divorced but with psychosocial support provided by the researcher team, who were also proficient counsellors, kept them together. Interestingly, all the four couples in which the index partner was a man remained together. It seems, therefore, that divorce following HIV-discordant results was influenced by the sex of the HIV-negative partner; in particular, HIV-negative men living in HIV-discordant relationships appeared less prepared to deal with HIV-discordant results.

Perspectives towards HIV-discordant results corresponded with widespread conceptions that men are expected to be the index partner. Study participants narrated that women were stigmatised, ridiculed or even abandoned for having sexual intercourse outside their marriage, while such behaviour was simply disregarded if the perpetrator was the man. HIV-negative men failed to deal with HIV-discordant results because they seemed unprepared and unwilling to live with a female partner who had been infected with HIV. HIV-negative women in HIV-discordant relationships were similarly troubled with HIV-discordant results but grudgingly accepted to remain living with their HIV-positive male partners. All the four HIV-negative women living in HIV-discordant relationships were not employed and expressed that they could not autonomously subsist economically without support from their husbands (Table 2; Q10). Thus, the economic position female partners contributed to their willingness to remain in a relationship despite the existing HIV risk. It important to highlight that data for this study was collected before the national policy shift towards universal test and treat in 2016. Frequently, wives (participants) noticed change in the behaviour of their husbands post-HIVST since partners are familiar with each other’s behaviour as described in Q11 in Table 2. The quote suggests a deterioration of the emotional state of the male partner and the inability for both sexual partners to collectively communicate experiences to manage test results.

Among individuals living in HIV-positive concordant couples, both male and female partners perceived that the male who was the index patient who infected the female also illustrated by the quote above. As a result, men were repeatedly blamed by their partners for introducing HIV into the relationship (Table 2; Q12, Q13 and Q14). Most often, men accepted the responsibility of introducing HIV into the relationship and shouldered the psychological burden of this blame. Men typically felt that it was impossible for their female partner to be the index patient by overtly declining the possibility that they could have been infected by their female partners. Most men were usually ready to admit that they were the responsible for infecting their female partner (Table 2; Q15 and Q16).

### Coping strategies used by self-tested couples

Individuals living in couples deployed several approaches to deal with and manage test-related adverse psychological effects following new HIV-positive or HIV-discordant self-test results. Strategies for coping with test-related adverse psychological effects were identifiable at two levels namely positive and negative coping mechanisms.

### Positive coping mechanisms for overcoming test-related adverse psychological effects

Several positive coping approaches were used to help couples navigate and overcome test-related adverse psychological effects.

#### Post-test psychosocial support

External support from the research team and religious establishments permitted partners to navigate their situation and successfully deal with test-related adverse psychological effects while at the same time reinvigorating trust within the relationship (Table 2; Q17). Even in discordant relationships, post-test counselling, especially given during confirmatory HIVST by the study team or at a routine health facility, helped to diffuse distrust within a relationship as described by an HIV-positive woman living in HIV-discordant relationship that survived marriage dissolution (see Table 2; Q18).

#### Positive behaviour change

Positive lifestyle adjustments of a distrusted partner expedited overcoming test-related adverse psychological effects among self-tested individuals living in couples. The outward display of virtuous behaviour that demonstrated some form of behaviour adjustment by a distrusted partner helped couples manage test-related psychological effects. The commonly cited behaviour change attributes included spending free time with a partner; not drinking alcohol; spending nights at home; coming home in good time; openness; remorse; active involvement in religious faith; having friends with good behaviour; and not engaging in adulterous sexual relationships. For example, a female partner testified a great transformation in the behaviour of her infected male partner post-HIVST in Q19 of Table 2. A positive change in the behaviour of a male partner helped to regenerate previously lost trust since this transformation appeared to be sincere (Table 2; Q20). Another example with a comparable pattern came from a female HIV-positive participant in HIV-discordant relationship where post-test counselling allowed the relationship to survive the adverse psychological effects introduced by HIV-discordant results. A change in the behaviour of the male partner and redeemed trust were attributed to post-test counselling provided by the research team (Table 2; Q21).

### Negative coping mechanisms for overcoming test-related adverse psychological effects

#### Questioning the authenticity of self-test results

Incompetence to navigate and deal with new HIV-positive and HIV-discordant self-test results repeatedly precipitated distrust towards HIVST approach itself as sexual partners questioned the authenticity of test results. Oral-fluid samples, instead of the conventional blood-based samples, became a valid basis for misconstruing self-test results. As a result, some participants failed to establish an association between HIV and oral fluids since it was a common knowledge within the study setting that HIV is found in the blood (Table 2; Q22). Both the HIV-positive and HIV-negative partners in HIV-discordant relationships perceived that the HIV-negative result of the uninfected partner was not a true reflection of the authentic HIV serostatus. Distrust in test-results introduced tension within uninfected people at two levels. Firstly, distrust towards the ability of HIV self-test kits to accurately diagnose HIV, and secondly, distrust in an individual’s competence to execute the essential self-testing procedures appropriately. Quote 23 in Table 2 illustrate a possibility for human error in carrying out a self-test. In this specific case, the female partner obtained the test-kits and the accompanying self-testing instructions through the male partner. The participants stated that she was distrusting self-test results when the true meaning of the word ‘distrust’ in the quote vividly demonstrate that she was allowing the results to gradually settle in. Interestingly, questioning of the authenticity of self-test results seemed useful in promoting linkage to confirmatory testing at a routine facility. Out of the 28 HIV-positive individuals who participated in this study, all the 21 new HIV-positive participants confirmed their self-test results at the clinic.

#### Misconceptions about test results

Failure to deal with HIV-discordant results was visible within the emerging post-test misconceptions for explaining the inconsistencies in test outcomes. HIV-negative individuals in HIV-discordant relationships presumed that they had already contracted HIV, and that HIVST occurred during the window period, a time when HIV test-kits are not capable of detecting HIV. Participants felt that the HIV virus was merely hibernating or hiding in their body but was expected to surface with time (see Table 2; Q24). Interestingly, one couple claimed that excessive consumption of strong alcohol rendered HIV less detectable. A male partner who tested HIV-negative and who later abandoned his HIV-positive partner conveyed misconceptions about a perceived link between alcohol misuse and HIV detection (Table 2; Q25).

#### Post-test blame

Depending on test results, sexual partners frequently held the infected partner (in discordant relationships) or a partner with a previous record of infidelity (for HIV-positive concordant relationships, mostly the male partner) accountable for introducing HIV into the relationship. For those in HIV-discordant relationships, blame was directed towards the infected partner. In some cases, the uninfected partner held someone else responsible for infecting their partner with HIV as a way of diverting intentionality from one’s partner. Shifting the culpability to a third party or ‘othering’ a source of infection was largely intended to demonstrate that HIV was not acquired through extramarital sex and to feel less guilty for infecting the other partner (Table 2; Q26). For those in HIV-concordant relationships, men were regularly accused of introducing HIV into the relationship. The quote below described how a woman vented her frustrations on her husband when she self-tested HIV-positive (Table 2; Q27). Among individuals living in HIV-discordant couples, blame was activated by an HIV-negative partner’s inquisitiveness to understand both causality and intentionality resulting into the other partner being infected with HIV. There seemed to be little or no difference about blame in relationships between those who self-tested either as couples or as individuals. Within the study context, partners were often unsupported in addressing issues of blame.

## Discussion

Findings from this study have demonstrated evidence of post-test adverse psychological effects and described coping mechanisms amongst self-tested individuals living in couples following new HIV-positive and HIV-discordant test results. Heightened levels of adverse psychological effects are common amongst individuals recently diagnosed with HIV who often exhibit several symptoms including suicidal thoughts, impaired well-being, fear of reaction from family members, fear of social disgrace, depression, denial, weight loss and anxiety [30,31]. Adverse psychological effects after HIV-positive results such as a feeling of hopelessness, shock, crying and blaming oneself were also reported within PMTCT programme from rural Malawi [32]. All new HIV-positive participants who experienced test-related adverse psychological effects in this study confirmed their HIV statuses through routine HTS within 7 days of testing where they received relevant post-test counselling and linkage information. However, insufficient information and support was provided to HIV-negative individuals who had an HIV-positive partner partly because of the lack of knowledge for managing complex relationship dynamics emerging because of serodiscordant results and their persistent pressure to meet cluster-level uptake targets for the trial [33]. To deal with this problem, we included HIV-discordant management in the training curriculum of HIVST providers in subsequent large-scale HIV self-testing implementation programmes [34]. A positive but an unanticipated finding was that scepticism and distrust towards authenticity of HIV-positive result from an oral-fluid based test kit encouraged self-tested individual to get a second opinion by confirming test results using conventional HIV-testing approaches at a facility. Psychological refusal to accept one’s HIV diagnosis has been reported to be prevalent especially where individuals perceive that their state of health is excellent [35]. This unanticipated result provides certain amount of hope that individuals testing HIV-positive through HIVST may have a drive to visit healthcare facility to confirm their results and subsequently link to follow-up HIV care.

Our findings have demonstrated physical and emotional unpreparedness to deal with new HIV-positive and HIV-discordant results obtained through HIVST among individuals living in heterosexual couples [36] because adverse psychological effects mostly occurred in first-time HIV-positive individuals and those in HIV-discordant couples. Thus, self-testers obtaining HIV-positive or HIV-discordant results for the first-time are less equipped to manage HIV-positive and HIV-discordant test results [37]. Researchers noted that the counselling provided to self-tested individuals mostly emphasised on the individual with little effort to understand the specific social context or social position of the individual. Where both partners tested positive, the burden of adverse psychological effects seemed to be smaller suggesting an enhanced coping competency for concordant HIV-positive couples [37]. To manage complex adverse psychological effects emerging because of new HIV-positive or HIV-discordant test result, heterosexual couples engaged both positive and negative coping strategies; the most significant being behaviour change, post-test psychosocial support, blame, inability to accept test results and misconceptions about HIV-discordant results. Use of diverse coping mechanisms may suggest that the post-test information or counselling provided did not sufficiently enable individuals couples to effectively navigate and manage test-related adverse psychological effects. The content and quality of support and information provided to individuals living in couples should therefore transcend beyond managing HIV infection towards equipping partners with skills of collectively managing undesirable relationship dynamics emerging as a result on HIV test results [38].

It is apparent from our data that an HIV diagnosis remains a shocking experience for to many including those living in heterosexual relationships despite the expanded availability of ART and HIV related information [39,40]. These findings also confirm the amplified anxieties especially among individuals living in HIV-discordant couples when compared to those in concordant HIV-positive couples and supports evidence of a high preference towards serosorting among people living with HIV [41]. Since HIV-discordancy digressed from the anticipated concordant results, it created disorder and disequilibrium between couples and undermined both individual and collective coping competency. In contrast, when couples experienced HIV-positive concordant results, they seemed less worried since the results created a sense of equilibrium within the relationship dynamic because of a shared HIV serostatus. These results are not different with those reported from facility-based testing since test-related reactions are mostly directed towards test results and not the mode of testing [42,43].

Post-test support from a third-party such as CCs acted as an important verbal encouragement in enabling sexual partners to manage their physical and emotional state and to cope with test results. Without any external support, the adverse psychological effects described here negatively influenced partners’ collective abilities to navigate and deal with new HIV-positive and HIV-discordant self-test results suggesting the importance of aligning post-test counselling messaging to the specific needs of couples. This is the reason why the 2018 Malawi HIV Self-testing Operational guidelines advocate for pre-test information provision to individuals intending to use a self-test kit and strongly emphasises referral of all HIV-positive self-test results to confirmatory testing using the national algorithm which contains post-test counselling component [44]. Worth noting is that the HIVST operational guidelines recognises both facility-based and community-based HIV self-testing delivery models using both primary and secondary distribution approaches [44].

Poverty and economic dependence–a prominent feature among our female participants–influenced women to be more accepting of male partners’ infidelity and correspondingly to deal with knowledge of having acquired HIV from their male partner [45]. Having a new HIV-positive or HIV-discordant self-test result meant that women found themselves at crossroads of decision-making process regarding the future of their marriage as they reflected on multiple things including own social position attached to marriage, economic welfare of themselves and their children against their current HIV results, the need safeguard own health and questionable morality of the male partner. By carefully analysing such complex and conflicting priorities while in an economically disadvantaged position, women felt powerless to opt out of the relationship where the male partner tested HIV-positive or was the index partner and subsequently forfeited own ability to manage potential risk [46]. In contrast, men seemed less able to cope with HIV-discordant results especially where the female partner was an index partner because HIV infection was directly interpreted to be linked to infidelity. In other words, HIV-negative men used HIV-positive status of the wives as the tool to identify or confirm infidelity [46]. Men felt that when their wife had sexual relations outside marriage, it was emasculating and demoted them to a status of a ‘lesser man’. In this context, men considered women’s sexual misconduct to be damaging to their ego and their ability to control the woman’s sexuality. As a collective unit, sexual partners had a diminished coping ability to manage test-related adverse psychological effects and this affected couples’ ability to effectively adapt after HIV-positive or HIV-discordant results.

The main methodological limitation for this analysis was a lack of data in this study comparing adverse psychological effects from HIVST versus facility-based HIV testing. This comparison would have been useful for deducing whether the dynamics after HIVST were comparable to those of routine HTC. Nevertheless, the existing body of literature on post-test dynamics after facility-based HTC provided a platform for this comparison [42,43]. This qualitative design did not permit analysis of the levels of post-test adverse psychological effects and the influence of relationship duration on coping abilities following concordant HIV-positive and HIV-discordant results.

## Conclusion

This paper has qualitatively discussed adverse psychological effects following new HIV-positive and HIV-discordant self-test results occurring amongst individuals living in couples. Self-tested participants living in couples seemed to lack both the individual and collective abilities to manage adverse psychological effects emerging after new HIV-positive and HIV-discordant results. This inability seemed to be amplified by the differing social and economic positions of men and women. Individuals testing HIV-positive or obtaining HIV-discordant results for the first-time within the context of a relationship undergo unique psychological challenges, as they must deal with the knowledge of an HIV infection at the intrapersonal level, but manage outcomes of having this information at the interpersonal level. Our findings suggest that the availability of post-test psychosocial support directed at management of relationship dynamics may be beneficial in reinforcing both individual and collective coping ability and resilience towards HIV-positive and HIV-discordant results. However, more research is required to quantify the magnitude of the deficiency in coping competency among heterosexual couples following an HIV self-test.
